# Supplementary material for: Simplification of networks by conserving path diversity and minimisation of the search information
Source: Sci Rep. 2020 Nov 5;10:19150. doi: 10.1038/s41598-020-75741-y (PMC7644697; doi:10.1038/s41598-020-75741-y)
Supplement: Supplementary file 1 — Supplementary Information. [file 41598_2020_75741_MOESM1_ESM.pdf]

Supplementary Information

Simplification of networks by conserving path diversity and minimisation of the search information.

H. Yin, R. G. Clegg and R. J. Mondragón

All the networks datasets used here can be found in KONECT (the Koblenz Network Collection http://konect.uni-koblenz.de/) and ICON (The Colorado Index of Complex Networks https://icon.colorado.edu/#!/).

Search information for some real networks and their simplification

Table 1 shows the search information H_o for several real networks with N_o nodes and its maximal and minimal search information when the networks are simplified. The maxima search information H_{max} is for a skeleton with N_{max} nodes and the minimal search information is H_{min} for a skeleton with N_{min} nodes.

Network	N_o	H_o	N_{max}	H_{max}	N_{min}	H_{min}
Bison	26	2904	26	2904	26	2904
Cattle	28	3415	27	3078	27	3078
Hens	32	4915	32	4915	32	4915
Karate	34	6061	31	5078	29	4233
USA	49	18093	46	15075	46	15075
Dolphins	62	26082	39	8761	35	6469
Polbooks	105	89776	93	66496	92	63529
D.Copperfiled	112	86869	66	26263	58	19369
Football	115	94428	83	44867	74	34081
FloEcoWet	128	112356	125	106205	124	103914
FloEcoDry	128	112210	125	106067	124	103778
JazzMusicians	198	349259	191	319189	191	319189
C.elegant	279	685719	240	489999	233	456838
TFL	369	1508840	58	120362	51	31382
Network Scientist	379	2615736	343	1983709	343	1983707
Airport	500	2930302	399	1777967	398	1768273
Taro	22	1909	16	1003	13	542
Residence	217	388220	184	264908	178	245237
Swedish	15	774	6	93	6	93
LesMis	77	46293	57	22726	57	22724
JPR	92	56563	72	31687	66	26048
Teacher	60	22117	26	4052	19	1769
Trainbomb	64	30353	50	16750	50	16139
Friendship	22	2153	20	1638	20	1638
Ecoli	329	1230895	102	142646	93	87239
Gamethrones	107	94989	87	57223	87	57174
New Spain	224	519626	31	112130	30	13937
London Gang	54	17333	53	16533	53	16533
Massachusetts	74	42881	52	19310	48	15126
Siren	44	12429	29	5646	29	5646
911terrorist	62	28781	48	15525	48	15036
Mexican	35	6067	32	4960	32	4898
Blumenaudrug	75	35567	43	10141	36	6698
Strike	24	2804	16	1149	16	1067
Grassland	75	42988	41	12202	40	10095
Rhodesbomb	22	2051	18	1241	18	1241

Korea	33	6456	24	2927	24	2927
Literary	35	5588	22	2035	16	897
Heroin	38	7453	29	4184	28	3712
Gang	29	4021	23	2217	23	2217
Saltmarsh	128	115299	116	93734	109	79569
SEP1999	56	22136	26	3985	26	3586
Attiro	59	21392	41	9847	34	6014
Yeast	662	5455250	233	558288	215	463329
Sanjuansur	75	38830	49	14908	42	10105
KBN	519	2818170	168	246851	138	159801
Anaheim	416	2290732	147	235141	131	170660
Russiatrade	39	7471	18	1761	13	673
Mali	36	7838	21	2138	21	2138
Cherowitzo	60	21856	33	5838	29	3979
Italiangang	65	32393	43	12405	43	12380
Prison	67	28083	38	8267	32	5217
Basketball	56	18978	36	6865	30	4276
Copilot	48	12988	41	9165	39	7856
Stmarks	54	16426	51	14298	51	14225
Drugnet	193	403421	55	34545	52	20839
Sawmill	36	7056	26	3334	23	2430
Maspalomas	24	2413	22	2029	22	1978
Parisfootball	35	5911	26	2980	26	2904

Table 1

The path diversity of different network simplification methods

Table 2 shows the evaluation of the cyclomatic number under different clustering algorithms. The cyclomatic number is $C = L - N + P$ where L is the number of links, N the number of nodes and P the number of connected components.

Network	C_O	C_{TC}	C_{Lovian}	C_{FG}	C_{IM}	C_{WT}	C_{BC}
Karate	45 (34,78)	45 (29,73)	27 (34,57)	28 (34,59)	33 (34,64)	17 (34,46)	25 (34,54)
Dolphins	98 (62,159)	98 (38,135)	63 (62,120)	73 (62,131)	66 (62,116)	73 (62,131)	70 (62,127)
Polbooks	337 (105,441)	337 (92,428)	299 (105,400)	302 (105,403)	278 (105,377)	302 (105,403)	299 (105,399)
D.Copperfield	314 (112,425)	314 (62,375)	93 (112,198)	100 (112,205)	311 (112,421)	124 (112,211)	75 (112,118)
Football	499 (115,613)	499 (78,576)	338 (115,444)	339 (115,448)	320 (115,423)	327 (115,432)	330 (115,435)
JazzMusicians	2545 (198,2742)	2545 (191,2735)	1812 (198,2006)	1943 (198,2137)	2357 (198,2548)	1967 (198,2154)	1786 (198,1945)
C.elegans	2009 (279,2287)	2009 (239,2247)	1223 (279,1498)	1488 (279,1763)	1381 (279,1662)	1422 (279,1698)	1518 (279,1789)
TfL	62 (369,430)	62 (54,115)	34 (369,384)	37 (369,387)	26 (369,339)	36 (369,360)	30 (369,379)
Network Scientist	536 (379,914)	536 (343,878)	488 (379,849)	495 (379,855)	432 (379,770)	493 (379,846)	489 (379,850)
Airport	2481 (500,2980)	2481 (399,2879)	1603 (500,2090)	1573 (500,2062)	2247 (500,2716)	1905 (500,2364)	2400 (500,2792)

Table 2. The path diversity of simplified networks obtained from tree-contraction method and 5 different graph partitioning methods. The upper row of C_O is the cyclomatic number of the original networks. C_{TC}, C_{Lovian}, C_{FG}, C_{IM}, C_{WT} and C_{BC} are the cyclomatic number of the simplified networks obtained from the different simplification methods. The numbers in brackets are the number of nodes and edges between clusters.

Search information for some large networks and their simplifications

Table 3 shows the search information H_o for several large networks with N_o nodes and its maximal and minimal search information when the networks are simplified. $H_{skeletonmax}$ is the maximal search information of the skeleton with $N_{skeletonmax}$ nodes and $H_{skeletonmin}$ is the minimal search information of the skeleton with $N_{skeletonmin}$ nodes.

Network	N_o	H_o	$N_{skeletonmax}$	$H_{skeletonmax}$	$N_{skeletonmin}$	$H_{skeletonmin}$
Internet	11175	1898544710	5224	405367311	5192	399437235
AS	10901	2079970369	5744	506240512	5720	501864031
BGP	17447	5600982163	8769	1239334130	8728	1226782718
Caida	26476	13635729216	11264	2103763543	11211	2082011965
Chess	7116	792010798	2989	119923171	2951	116773785
Chicago	12980	7912207191	4395	662173757	4369	605898315
Google	23614	11238819758	6721	741892468	6717	729417488
HostPathogen	10862	2067554579	2253	70718006	2205	67440512
Interactome	5583	473240887	1248	18630363	1224	17819455
PGP	10681	2693004566	4205	325075319	4191	321274251
SisterCity	10321	1872213563	1081	14204962	1041	13097524
Skitter	9201	1333980676	5603	456250412	5587	453370967

Table 3. Search Information
